# Invasive clonal plants possess greater capacity for division of labor than natives in high patch contrast environments

**DOI:** 10.3389/fpls.2023.1210070

**Published:** 2023-07-10

**Authors:** Jin Zhang, Wen-Hua You, Ning-Ning Li, Dao-Lin Du

**Affiliations:** Institute of the Environment and Ecology, College of the Environment and Safety Engineering, Jiangsu University, Zhenjiang, China

**Keywords:** plant invasion, clonal plants, clonal integration, heterogeneity, root to shoot ratio

## Abstract

Invasion success of clonal plants is closely related to their unique clonal life history, and clonal division of labor is a crucial clonal trait. However, so far, it is unclear whether invasive alien clonal species generally possess a greater capacity for division of labor than native species and whether this pattern is affected by environmental conditions. To test whether patch contrast affects the differences in the capacity for division of labor between invasive alien and native clonal plants, we selected five pairs of exotic invasive and native clonal plant species that are congeneric and co-occurring in China as experimental materials. We grew the clonal fragment pairs of these invasive and native plants under high, low, or no contrast of reciprocal patchiness of light and nutrient, respectively, with ramet connections either severed (division of labor prevented) or kept intact (division of labor allowed). The results showed that connection significantly decreased the proportion of biomass allocated to roots in distal (younger) ramets, whereas it increased in proximal (older) ramets of all studied plants under high -contrast treatments. This clear pattern strongly indicated the occurrence of division of labor. Furthermore, the connection had a more pronounced effect on the pattern of biomass allocation of invasive alien plants, resulting in a greater increase in biomass for invasive alien plants compared to native plants. These findings suggest that the invasive alien plants possess a greater capacity for division of labor, which may confer a competitive advantage to them over natives, thus facilitating their invasion success in some heterogeneous habitats such as forest edges where light and soil nutrients show a high negative correlation.

## Introduction

Biological invasions are reported to be a major threat to global biodiversity and can cause serious economic and ecological damage (Diagne et al., 2021; Li et al., 2022). Many studies have been conducted to identify and understand the mechanisms underlying the process of invasion, and some inherent traits associated with clonal growth are widely recognized as critical determinants contributing to plant invasiveness (Song et al., 2013; Roiloa, 2019). The plausibility of this argument rests on the fact that many of the most problematic invasive plant species exhibit clonal propagation (Cadotte et al., 2006; Roiloa, 2019). Furthermore, recent studies have demonstrated that clonal- introduced plants decrease the native richness to a greater extent than non-clonal introduced plants worldwide (Vilà et al., 2015; Franklin et al., 2021).

A crucial clonal trait is the capacity for division of labor mediated by physiological integration and driven by source–sink relationships (Hutchings and Wijesinghe, 1997; Xi et al., 2019). It is defined as the specialization of resource uptake between independent parts of the clonal plants (Stuefer, 1998; Roiloa et al., 2016). In nature, resources essential for plant growth and survival, such as light, water, and nutrients, are typically distributed unevenly (Jackson and Caldwell, 1993; Peipoch et al., 2016). By means of vegetative growth and reproduction, clonal plants have the ability to occupy extensive areas, thus increasing their potential to encounter environmental heterogeneity (Stuefer and Hutchings, 1994, Marshall, 1999). In some environments, the availability of two resources may be negatively correlated, especially when high availability of one resource is accompanied by a decline in the other (Friedman and Alpert, 1991; Struefer et al., 1996). For instance, nitrogen-fixing shrubs could increase the effectiveness of soil N but reduce the level of light under their canopy (Friedman and Alpert, 1991; Roiloa et al., 2007; Wang et al., 2011). The division of labor allows each ramet to capture locally abundant resources, as resource uptake is more economical in more resource-rich patches, and subsequent reciprocal transfer of resources between ramets should improve the performance of the whole clone (Stuefer et al., 1996; Huang et al., 2018; Roiloa, 2019). Numerous studies have shown that the negative correlation between the spatial distribution of different basic resources induces a division of labor and that the division of labor improves the performance of clonal plants (Friedman and Alpert, 1991; Wang et al., 2016; Huang et al., 2018; Lin et al., 2018).

Contrast, which refers to the extent of the relative variation in resource availability between patches or between a patch and its surrounding matrix, constitutes one aspect of environmental heterogeneity (Struefer et al., 1996). Theoretical studies predict that greater patch contrast may lead to a stronger division of labor (Stuefer et al., 1998; Magyar et al., 2007). This is also supported by experimental evidence. For example, Wang et al. (2011) found that the division of labor occurred only when the patch contrast exceeded a threshold in an environment where soil nutrients and light were negatively correlated. Roiloa et al. (2007) showed that clones from habitats with greater patch contrast had a stronger division of labor than those from more homogeneous habitats. More recently, a study by Roiloa et al. (2019) found that the highly invasive exotic clonal species *Carpobrotus edulis* exhibited greater division of labor relative to the exotic non-invasive clonal species *Carpobrotus chilensis.* This suggests that the division of labor may be a feature of the correlation between clonal growth and plant invasion (Roiloa et al., 2019). However, to date, little is known about whether the capacity for division of labor between invasive alien clonal plants and native plants differs in certain regions and how it is affected by patch contrast.

In the present experiment, to avoid large differences between invasive and native species in their habitat preferences and phylogenetic relatedness (Felsenstein, 1985), we selected five pairs of congeneric and co-occurring invasive and native clonal plant species to serve as experimental material. We grew the clonal fragment pairs of these invasive and native plants under high, low, or no contrast of reciprocal patchiness of light and nutrient, respectively, and with ramet connections either severed (division of labor prevented) or kept intact (division of labor allowed). We predicted that invasive plants have greater capacity for division of labor than natives. Based on the theoretical studies, we further predicted that the difference in the capacity for division of labor would be greater under higher patch contrast.

## Materials and methods

### Species selection and cultivation

We chose five pairs of asexual clonal plants, three of which were stoloniferous and the other two were rhizomes, as described in **Table 1**. In each pair, one species is an invasive alien species, and the other is a common native species in China that is co-occurring with the invasive alien species in the wild. We opted for species within the same family (or genus) in order to elucidate the phylogenetic correlation between the two species within each pair. All plants used were collected from the field in Jiangsu Province or Guangdong Province (China). To enhance the probability of collecting plant material from different genotypes (genes), we obtained multiple fragments of each species from various locations separated by over 500 m. Then, the collected fragments were propagated asexually in a greenhouse at Jiangsu University in Zhenjiang, Jiangsu Province, China. In April 2022, 36 similarly sized pairs of plants of each species were selected for the following experiment, each pair consisting of two rooted, similarly sized ramets interconnected by a single stolon or rhizome internode.

**Table 1:** Clonal plant species used in the experiment.

Species	Family	Origin	Native range	Clonal organ	Typical habitat
***Sphagneticola trilobata* (L.) Pruski**	Asteraceae	Invasive alien	North and South America	Stolon	Moist grasslands, edges of canals, roadsides
***Sphagneticola calendulacea* (L.) Pruski**	Asteraceae	Native	Asia	Stolon	Moist grasslands, edges of canals, crop fields, roadsides
***Alternanthera*** ***philoxeroides* (Mart.) Griseb**	Amaranthaceae	Invasive alien	South America	Stolon	Wetlands, canals, nearby fields
***Alternanthera sessilis*** **(L.) DC**	Amaranthaceae	Native	Asia, Africa	Stolon	Wetlands, other moist habitats
***Hydrocotyle verticillata* Thunb.**	Araliaceae	Invasive alien	North America, Europe	Stolon	Wetlands, other moist habitats
***Hydrocotyle sibthorpioides***	Araliaceae	Native	Asia	Stolon	Wetlands, other moist habitats
***Paspalum notatum* Flugge**	Poaceae	Invasive alien	North and South America	Rhizome	Roadsides and grasslands
***Paspalum orbiculare* (G. Forster) Hackel**	Poaceae	Native	Asia, Oceania	Rhizome	Roadsides, other moist habitats
***Paspalum virgatum* L.**	Poaceae	Invasive alien	South America	Rhizome	Moist grasslands
***Paspalum distichum* L.**	Poaceae	Native	Tropics and subtropics of Asia, America	Rhizome	Moist grasslands

Origin and habitat information are based on the Flora of China (www.iplant.cn), Scientific Database of China Plant Species (DCP) (http://www.plants.csdb.cn/eflora), and other reference (Wang et al. (2017).

### Experimental design

The experiment took place in the greenhouse at Jiangsu University. In late April 2022, we transplanted each pair of ramets into two plastic pots measuring 120×88×188 mm (top bottom × bottom × height), with a small 2×2cm opening at the top of each pot for the rhizome or stolon connecting the two ramets to pass through. The substrate consisted of a blend of river sand and yellow-brown soil in a 1:1 ratio by volume, with a very low nutrient concentration (Xi et al., 2019).

After a recovery period of approximately 1 week, we conducted the experiment to assess the impacts of species origin, intact stolon/rhizome, and patch contrast. We designated younger ramets growing in high light and low nutrient patches as distal ramets and older ramets growing in low light and high nutrient patches as proximal ramets. The connection between the two ramets was either severed in the middle (preventing division of labor) or kept intact (allowing division of labor).

The light and nutrient addition protocols for all fragments in the experiment are presented in **Table 2**. To create different patch contrast environments, we used polypropylene shade nets of varying shade intensities to cover the ramets, while controlling nutrient effectiveness through the use of different quality of slow-release fertilizers. For each combination of the experiment, we established six replicates, resulting in a total of 360 ramet pairs across 10 species. During the experimental period, regular watering was provided to support plant growth, and the average light intensity at noon was 1,200–1,400 µmol m^−2^ s^−1^, with a mean air temperature of 25°C–32 °C in the greenhouse. The experiment was conducted for 9 weeks and ended in early July 2022.

**Table 2:** Light exposures and nutrient concentrations applied to the ramets in three treatments with different patch contrasts (control, low, and high).

	Proximal		Distal	
Patch contrast	Light (% full sunlight)	Nutrient (g)	Light (% full sunlight)	Nutrient (g)
*Control*	55	0.5	55	0.5
*Low contrast*	40	0.7	70	0.3
*High contrast*	10	0.9	100	0.1

Notes: The fertilizer used is slow-release fertilizer, Osmocote^R^, N–P–K: 16–9–12.

### Measurements

We harvested the distal and proximal ramets in each pair of containers. The clonal fragments in each container were separated into below-ground (root) and above-ground (shoot) parts. Different plant parts were dried in an oven at 80°C for 72 h and then weighed to obtain the dried biomass.

### Statistical analysis

We used histograms and quantile–quantile plots to graphically check whether the residuals of all models were normally distributed. This was made using the *ggplot* function of the “ ggplot2” package (Wilkinsom, 2016) in R 4.2.0 (R Core Team, 2022). Data transformation was performed to satisfy normality if necessary. We analyzed the effect of treatments on the biomass and root to shoot ratio using a linear mixed model with the *lme* function from the R package “nlme” (Pinheiro et al., 2020). In these models, we used species origin (invasive vs. native), intact (stolon/rhizome remaining connected or severed), patch contrast (control vs. low contrast vs. high contrast), and their interaction as fixed factors. To account for the differences between species pairs and species, we included species nested within species pairs as random factors in our model. In addition, since the variance varies between species, we used the *varIdent* function of the “ nlme” package to allow each species to have a different variance structure (Pinheiro et al., 2020). The significance of fixed effects was assessed using likelihood ratio tests when comparing models with and without the effects of interest (Zuur et al., 2009). All analyses were performed using the free software R (version 4.2.0; R Development Core Team, 2022).

## Results

**Figure 1:**
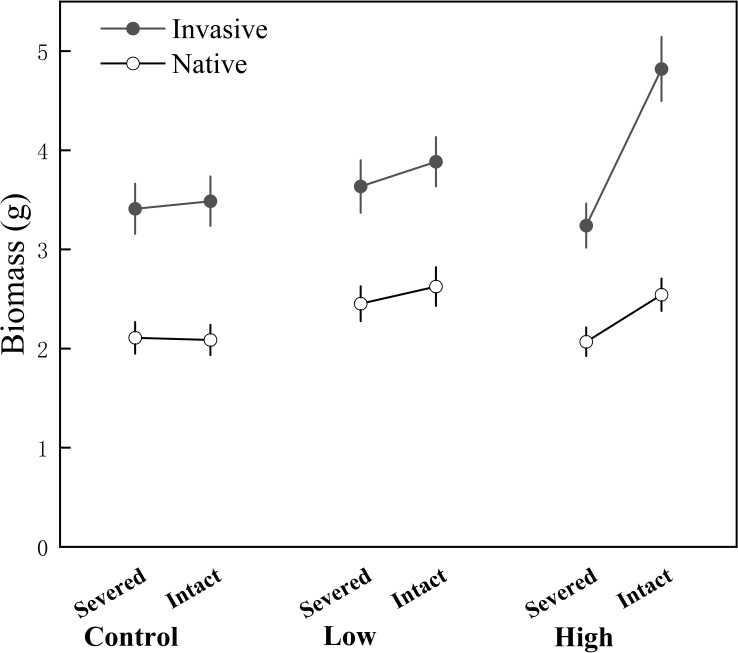
Biomass of the whole clone of the invasive alien and native clonal species when the clone was grown under high- contrast, low- contrast, and control treatments with connections between the proximal and distal ramets severed or remained intact. Values are means ± standard error (SE).

**Figure 2:**
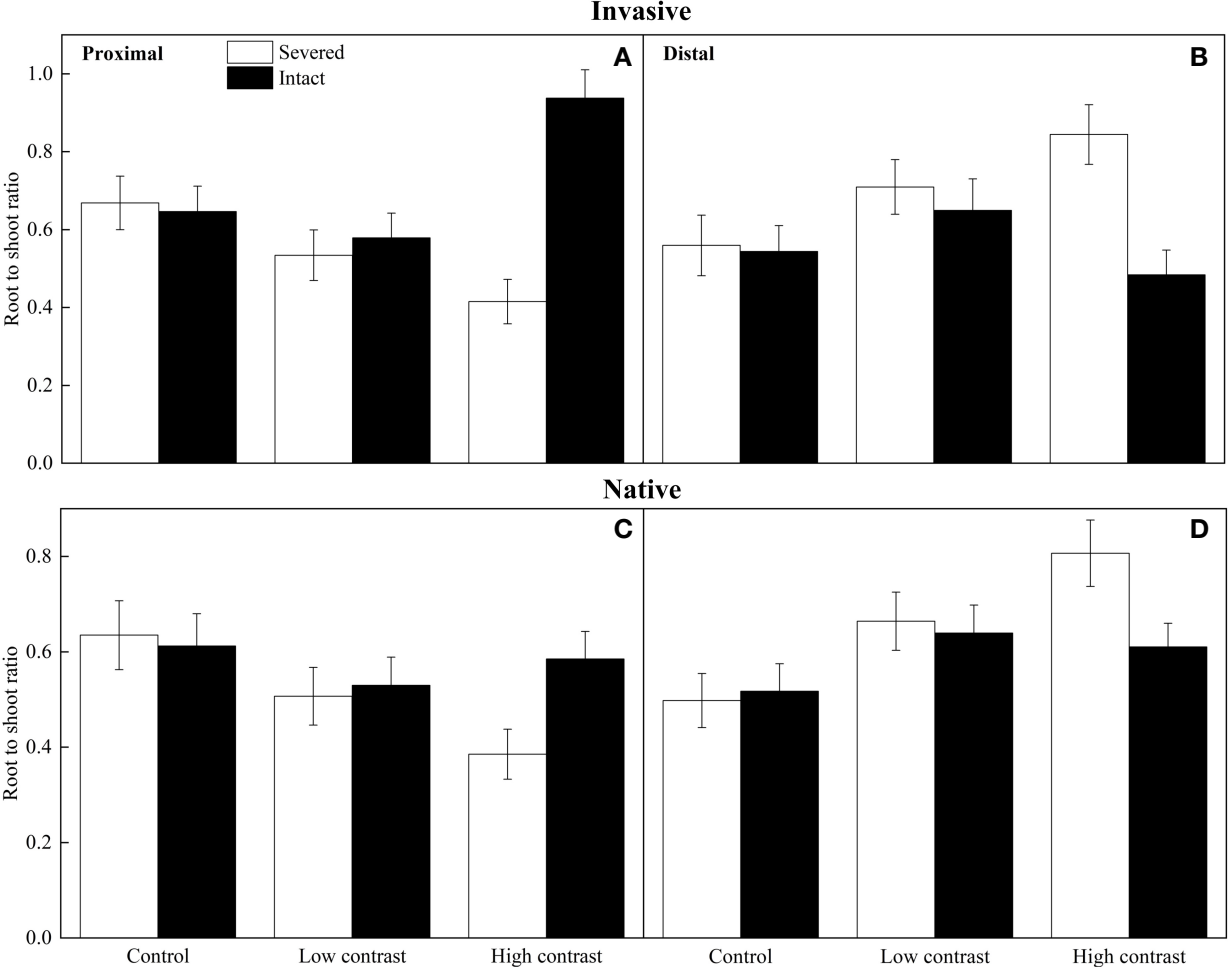
Effects of experimental treatments on the root to shoot ratio of proximal and distal ramets of the invasive alien **(A, B)** and native **(C, D)** clonal species. The data indicate the means ± SE.

Overall, invasive plants had a greater biomass than native plants (**Figure 1**). Connection (intact) significantly increased the root to shoot ratio of proximal ramets, whereas it decreased in distal ramets under high contrast, as indicated by significant intact × contrast interaction (**Table 3**; **Figure 2**). The effect of connection on the root to shoot ratio of proximal ramets was more significant in invasive plants than in native plants under high contrast (significant origin × intact × contrast interactions in **Table 3**; **Figures 2A, C** **)**. Similar results also occurred in the distal ramets (**Table 3**; **Figures 2B, D** **)**. Moreover, the connection greatly increased the total biomass (proximal biomass + distal biomass) of the whole clone under high contrast, especially for invasive species (significant origin × intact × contrast interactions in **Table 3**; **Figure 1**).

**Table 3:** Results of linear mixed models for effects of origin (invasive vs. native), patch contrast (control vs. low vs. high), and intact (connection vs. severed) on the total biomass of the whole clone and the root to shoot ratio of distal ramets and proximal ramets.

Model terms *Fixed effects*	df	Root to shoot ratio of distal ramets χ2 p		Root to shoot ratio of proximal ramets χ^2^ p		Total biomass χ^2^ p	
**Intact (I)**	1	**18.412**	**<0.001**	**31.712**	**<0.001**	**19.067**	**<0.001**
**Contrast (C)**	2	**47.807**	**<0.001**	**20.319**	**<0.001**	**15.135**	**0.005**
**Origin (O)**	1	0.687	0.407	**5.244**	**0.022**	**10.959**	**0.009**
**I × C**	2	**29.779**	**<0.001**	**69.174**	**<0.001**	**23.061**	**<0.001**
**I × O**	1	**6.024**	**0.014**	**5.525**	**0.018**	**6.648**	**0.01**
**C × O**	2	4.811	0.090	**12.032**	**0.002**	2.082	0.353
**I × C × O**	2	3.983	0.136	**8.375**	**0.015**	**6.248**	**0.044**
***Random effects***	N		**SD**		**SD**		**SD**
**Taxonomic pair**	5		0.552		0.155		0.833
**Species** identity^a^	10		0.066		0.019		0.382
**Residual**			0.428		0.099		0.546

Significant effects (p < 0.05) are shown in bold.

a The SDs shown in the table are for the alien species Wedelia trilobata (L.) Hitchc, and the SDs for all species are shown in **Supplementary Table S1**.

## Discussion

Not entirely consistent with the conjecture, our results suggested that in a patchy environment where light and nutrients were negatively correlated, division of labor occurred only under high- contrast treatments and that invasive alien plants had a greater capacity for division of labor than native plants. We can conclude that there is a difference in the capacity for division of labor between invasive alien and native clonal plants, but this difference is environmentally dependent.

No division of labor was observed in either the control or low- contrast treatments. One possible explanation is that division of labor is more likely to occur in patchy habitats where plant functions are limited due to resource scarcity. Previous studies suggest that when ramets are cultivated under optimal conditions, they do not exhibit adaptive responses to heterogeneous environment (Zhang and He, 2009). However, this explanation appears somewhat implausible when considering the findings of other researchers, who have demonstrated that the resource settings in our control and low-contrast treatments are indeed capable of reducing plant growth performance (Guo et al., 2011). In a patchy environment with reciprocal resource distribution, division of labor emerges as a vital mechanism for enhancing the performance of clonal plants. Each ramet concentrates its efforts on locally abundant resources. However, if the patch environment undergoes changes or the connections between ramets are severed, each ramet is then confronted with a scarcity of locally resources, at which point the division of labor may become detrimental (Stuefer et al., 1998; Magyar et al., 2007; Ikegami et al., 2008). Consequently, division of labor occurs exclusively when the growth of ramets faces significant constraints and the benefits derived from resource exchange far outweigh the associated costs. This is the result of a clone-wide cost–benefit tradeoff that is important for risk aversion, especially in disturbed environments (Wang et al., 2011).

In fact, there are also studies showing that clonal plants can significantly alter their distribution balance and show division of labor even in homogeneous environments (Dong et al., 2015; Xi et al., 2019). This phenomenon, known as “developmentally programmed division of labor,” is inherently governed by the plant’s internal developmental processes, independent of external environmental factors (Liu et al., 2016). In addition, the division of labor can be achieved not only by adjusting biomass allocation but also by regulating certain physiological functions and may be easier to express (Roiloa et al., 2007). Since physiological characteristics are more readily reversible compared to morphological traits, the risks to entire clones or clone fragments may be relatively lower (Wang et al., 2011). For example, Wang et al. (2011) found that all-lit connected ramets displayed significantly higher photosynthetic capacity than isolated ramets in three different patch contrast treatments of high, medium, and low, suggesting that the connected ramets are specialized for photosynthesis.

Connection significantly increased the proportion of biomass allocated to roots in proximal ramets and the proportion of biomass allocated to shoots in distal ramets under high- contrast treatments. This clear pattern strongly indicated the occurrence of division of labor. Additionally, we observed a substantial increase in the overall biomass of the entire clonal fragment when connected. These findings align with previous studies that have demonstrated the advantageous uptake and exchange of resources among clonal plants in heterogeneous resource environments, resulting in enhanced production efficiency, increased biomass, and improved fitness of the entire clonal system (You et al., 2013; You et al., 2014). Furthermore, our results highlighted a noteworthy distinction: the resource uptake specialization was significantly more pronounced in invasive alien plants compared to native plants. This suggests that invasive alien plants possesse a greater capacity for division of labor, which also leds to a greater increase in biomass for invasive species. The disparity in division of labor capabilities may confer a competitive advantage to invasive plants over native species, thereby facilitating their invasion.

Disregarding the division of labor, the invasive alien plants always had a greater biomass than native plants. Our results do not directly indicate that invasive plants are more competitive. In fact, a recent study by Wang et al. (2019) revealed that invasive clonal plants exhibited increased biomass production and vegetative reproduction when grown in the presence of interspecific competition compared to intraspecific competition, while the opposite was observed for native clones. This suggests that the invasive clonal plants are competitively superior to concurrently co-occurring native plants. The high intrinsic growth rates of the invasive plants may be the main driver of its high competitive ability (Zhang and van Kleunen, 2019).

One notable aspect to consider is that our study solely focused on the spatial heterogeneity of resource availability, disregarding temporal heterogeneity. Consequently, this study may not accurately reflect real-life habitat environments (Yu et al., 2018; Wang et al., 2021). A modeling study conducted by Magyar et al. (2007) indicated that the advantage of plasticity diminishes as the rate of environmental change intensifies, suggesting that we may be overestimating the benefits of division of labor. Therefore, future studies should strive to provide additional experimental evidence to further investigate this matter. Nonetheless, our empirical research suggests that invasive clonal plants have a stronger capacity for division of labor than native plants under high contrast.

Furthermore, Roiloa et al. (2016) found that the invasive clonal plant *C. edulis* could benefit more from division of labor in the invasion site compared to the native population through common garden experiments, suggesting that the division of labor, which positively contributes to the clonal growth and reproduction of clonal plants, has evolved rapidly and adaptively in the invasion area. Nevertheless, additional research is necessary to determine whether this adaptive evolution is a common occurrence among other invasive species.

In conclusion, our results showed that in a patchy environment where light and soil nutrients were highly negatively correlated, both exotic invasive clonal plants and native clonal plants in China were able to alleviate the pressure of resource scarcity and promote their own growth through division of labor. More importantly, invasive clonal plants had significantly greater capacity for clonal division of labor than native plants, which also brought them greater biomass increase. The difference in the capacity for clonal division of labor between exotic invasive clonal plants and native clonal plants may explain the success of invasion in certain habitats such as forest edge where light and soil nutrients show a high negative correlation.

## Data availability statement

The original contributions presented in the study are included in the article/**Supplementary Material**. Further inquiries can be directed to the corresponding author.

## Author contributions

JZ and W-HY conceived and designed the experiments. JZ and N-NL performed the experiments. W-HY and JZ analyzed the data. W-HY and D-LD contributed reagents, materials, and analysis tools. JZ, N-NL and W-HY wrote the manuscript. All authors contributed to the article and approved the submitted version.

## Supplementary material

Supplementary Table 1 Standard deviations of each invasive alien and native clonal species for each of the response variables in the models presented in **Table 3**.
