# Supplementary material for: Invasive clonal plants possess greater capacity for division of labor than natives in high patch contrast environments
Source: Front Plant Sci. 2023 Jul 10;14:1210070. doi: 10.3389/fpls.2023.1210070 (PMC10363633; doi:10.3389/fpls.2023.1210070)
Supplement: Supplementary Table 1 — Standard deviations of each invasive alien and native clonal species for each of the response variables in the models presented in Table 3. [file Table_1.docx]

**Table S1.** Standard deviations of each invasive alien and native clonal species for each of the response variables in the models presented in Tables 3.

|  | Root to shoot ratio of distal ramets | Root to shoot ratio of proximal ramets | Total biomass |
| --- | --- | --- | --- |
| *Wedelia trilobata* (L.) Hitchc | 0.066 | 0.019 | 0.382 |
| *Wedelia chinensis* (Osbeck.) Merrill | 0.055 | 0.018 | 0.209 |
| *Alternanthera philoxeroides* (Mart.) Griseb | 0.046 | 0.020 | 0.745 |
| *Alternanthera sessilis* (Linn.) DC | 0.043 | 0.015 | 0.448 |
| *Hydrocotyle vulgaris* L. | 0.060 | 0.017 | 0.678 |
| *Hydrocotyle sibthorpioides* | 0.055 | 0.015 | 0.149 |
| *Paspalum notatum* Flugge | 0.048 | 0.018 | 0.912 |
| *Paspalum orbiculare* Forst. | 0.042 | 0.016 | 0.683 |
| *Paspalum virgatum* L. | 0.055 | 0.019 | 0.711 |
| *Paspalum distichum* L. | 0.042 | 0.017 | 0.488 |
